# Hypertrophic and Dilated Cardiomyopathy-Associated Troponin T Mutations R130C and ΔK210 Oppositely Affect Length-Dependent Calcium Sensitivity of Force Generation

**DOI:** 10.3389/fphys.2020.00516

**Published:** 2020-06-03

**Authors:** Marcel Groen, Alfredo Jesus López-Dávila, Stefan Zittrich, Gabriele Pfitzer, Robert Stehle

**Affiliations:** ^1^Department of Neurology and Neurogeriatry, Johannes Wesling Medical Center, Ruhr-University Bochum, Bochum, Germany; ^2^Department of Molecular and Cell Physiology, Hannover Medical School, Hanover, Germany; ^3^Institute of Vegetative Physiology, University of Cologne, Cologne, Germany; ^4^Institute of Neurophysiology, University of Cologne, Cologne, Germany

**Keywords:** contractility, length dependent activation, thin filament regulation, cardiomyopathy, sarcomere length, force generation, troponin, calcium sensitivity

## Abstract

Length-dependent activation of calcium-dependent myocardial force generation provides the basis for the Frank-Starling mechanism. To directly compare the effects of mutations associated with hypertrophic cardiomyopathy and dilated cardiomyopathy, the native troponin complex in skinned trabecular fibers of guinea pigs was exchanged with recombinant heterotrimeric, human, cardiac troponin complexes containing different human cardiac troponin T subunits (hcTnT): hypertrophic cardiomyopathy-associated hcTnT^R130C^, dilated cardiomyopathy-associated hcTnT^Δ*K*210^ or the wild type hcTnT (hcTnT^WT^) serving as control. Force-calcium relations of exchanged fibers were explored at short fiber length defined as 110% of slack length (*L*_0_) and long fiber length defined as 125% of *L*_0_ (1.25 *L*_0_). At short fiber length (1.1 *L*_0_), calcium sensitivity of force generation expressed by −log [Ca^2+^] required for half-maximum force generation (pCa_50_) was highest for the hypertrophic cardiomyopathy-associated mutation R130C (5.657 ± 0.019), intermediate for the wild type control (5.580 ± 0.028) and lowest for the dilated cardiomyopathy-associated mutation ΔK210 (5.325 ± 0.038). Lengthening fibers from 1.1 *L*_0_ to 1.25 *L*_0_ increased calcium sensitivity in fibers containing hcTnT^R130C^ (delta-pCa_50_ = +0.030 ± 0.010), did not alter calcium sensitivity in the wild type control (delta-pCa_50_ = −0.001 ± 0.010), and decreased calcium sensitivity in fibers containing hcTnT^Δ*K*210^ (delta-pCa_50_ = −0.034 ± 0.013). Length-dependent activation indicated by the delta-pCa_50_ was highly significantly (*P* < 0.001) different between the two mutations. We hypothesize that primary effects of mutations on length-dependent activation contribute to the development of the diverging phenotypes in hypertrophic and dilated cardiomyopathy.

## Introduction

The Frank-Starling law describes the intrinsic ability of the heart ventricle to adapt the systolic stroke volume to the previous diastolic filling. One main reason for this ability is the increased calcium sensitivity of the stretched myocardium, reviewed in Hanft et al. (2008), de Tombe et al. (2010) and Ait Mou et al. (2015) and also termed length-dependent activation (LDA). LDA has been attributed to different mechanisms intrinsic to the sarcomere, involving changes in filament lattice spacing (Hanft et al., 2008), stretch-dependent Ca^2+^ regulation of troponin (Arteaga et al., 2000; Konhilas et al., 2003; Korte et al., 2012; Zhang et al., 2017), ordering of myosin head orientation (Farman et al., 2011), strain-sensing in titin (Millman and Irving, 1988; Ait Mou et al., 2015; Ait-Mou et al., 2016; Linke, 2018), and the communication between these mechanisms (Ait-Mou et al., 2016; Zhang et al., 2017).

Mutations in proteins of the sarcomere are associated with hypertrophic cardiomyopathy (HCM) and dilated cardiomyopathy (DCM). HCM and DCM mutations can occur in the same protein like in human cardiac troponin T (hcTnT) (Kamisago et al., 2000; Marston and Hodgkinson, 2001; Song et al., 2005; Lu et al., 2013). Cardiac troponin T (cTnT) transfers the Ca^2+^ binding event from cardiac troponin C (cTnC) to troponin I (cTnI) and tropomyosin (Tm) [recently reviewed in (Marston and Zamora, 2020)]. Previous studies investigating individual mutations in these regulatory proteins associated with HCM and DCM either mostly reported negative or no consequence of mutations on LDA (Li et al., 2013; Sequeira et al., 2013; Mickelson and Chandra, 2017; Reda and Chandra, 2018, 2019).

Hypertrophic cardiomyopathy and DCM differ not only as to heart morphology but also to the type of cardiac dysfunction and histology. Diastolic dysfunction and cardiomyocyte disarray is typical for HCM whereas systolic function and shape of cardiomyocytes are often normal in HCM. In contrast, severe systolic dysfunction and thinned cardiomyocytes are typical features of DCM. HCM mutations mostly increase calcium sensitivity of contraction while DCM mutations mostly decrease it (Robinson et al., 2002, 2007; Mirza et al., 2005; Willott et al., 2010; Kalyva et al., 2014). Besides the direct effect of mutations on calcium sensitivity, also their effect on posttranslational modification of regulatory proteins correlates with the type of cardiomyopathy (Sfichi-Duke et al., 2010; Lu et al., 2013; Memo et al., 2013; Messer et al., 2016). However, to the best of our knowledge, no study so far examined the effects of HCM and DCM-associated mutations on LDA within the same experiment, keeping identical conditions among control, HCM and DCM mutant.

To test if there is any difference in the effects of HCM- and DCM-associated mutations on mechanical parameters, in particular on LDA, we exchanged the native troponin complex in skinned fibers dissected from papillary muscle of the left ventricle of the guinea pig by recombinant human heterotrimeric troponin complexes (hcTn) containing different recombinant hcTnT: either hcTnT with the HCM-associated exchange of arginine-130 to cysteine (hcTnT^R130C^), hcTnT with the DCM-associated deletion of lysine-210 (hcTnT^Δ*K*210^), or wild type hcTnT as control. Fibers incorporated with the two mutations exhibited highly significant different and opposite response of calcium sensitivity to lengthening, i.e., calcium sensitization for the HCM and calcium desensitization for the DCM mutation. These findings suggest that the HCM and DCM mutation might exert opposite primary effects on the Frank-Starling mechanism.

## Materials and Methods

### Skinned Fiber Preparation and Mechanical Setup

Skinned fibers were dissected from the left ventricular papillary muscles of the guinea pig (Stehle et al., 2002) and stored for up to 60 h at 0°C in skinning solution containing 5 mM KH_2_PO_4_, 5 mM Na-acide, 3 mM magnesium acetate, 5 mM K_2_EGTA, 3 mM Na_2_MgATP, 47 mM sodium creatine phosphate, 2 mM dithiothreitol (DTT), 0.2 mM 4-(2-aminoethyl)benzenesulfonyl-fluoride (AEBSF), 10 μM leupeptin, 10 μM antipain, 5 mg/l aprotinin. Skinned fibers were mounted in skinning solution in the mechanical setup between a force transducer (KG7A) with bridge-amplifier DUBAM 7C (Scientific Instruments, Heidelberg, Germany) and a fixed clamp. After mounting, fibers were stretched by 10% of their slack length *L*_0_ to 1.1 *L*_0_.

### Troponin Exchange

The three subunits of hcTn, i.e., hcTnC, hcTnT, and hcTnI were separately expressed in *Escherichia coli* and isolated as described previously (Kruger et al., 2003). For the exchange of the endogenous guinea pig cTn for the exogenous recombinant human cTn, the fibers were incubated in the mechanical setup at 10°C for 15 min in exchange buffer (in mmol/L): 132 NaCl, 5 KCl, 1 MgCl_2_, 10 Tris, 5 EGTA, 1 NaN_3_, pH 7.1 (20°C) followed by incubation in the same buffer containing in addition 3 mg/ml hcTn for 180 min at 20°C (Neulen et al., 2007).

The exchange of the endogenous guinea pig cTn (gcTn) for the exogenous hcTn was probed by 12.5% SDS-PAGE and visualizing proteins by Commassie-R250-staining (Solzin et al., 2007). Guinea pig cTnI (gcTnI) contains one more amino acid and migrates less than hcTnI on the gel (Supplementary Figure S1 in Supplementary Material). Exchange efficiency was defined by the ratio of hcTnI intensity per total intensity of hcTnI and gcTnI and quantified using Phoretix-1 as illustrated in Supplementary Figure S1.

### Force-pCa Relations

Force-pCa relations were measured using mixtures of Ca^2+^-buffered activating and relaxing solutions containing 3 mM (CaCl_2_)K_2_EGTA (activating solution, pCa 4.7) or 3 mM K_4_Cl_2_EGTA (relaxing solution, pCa 7), 10 mM imidazole, 10 mM Na_2_MgATP, 3 mM MgCl_2_, 32.7 mM sodium creatine phosphate, 2 mM DTT, pH 7.0, μ = 178 mM. To ensure saturation of free Ca^2+^ concentration at all conditions, an extra activation solution (pCa 4.28) was prepared by adding 3 mL 60 mM CaCl_2_ per 100 mL activating solution. Experimental temperature was 10°C.

Force–pCa relations were fitted by sigmoidal Hill equation: *F*_norm_ = 1 + 10^(pCa50 – pCa)nH^, where *F*_norm_ is the force at pCa = −log [Ca^2+^]/M normalized to maximum force at pCa 4.28, pCa_50_ is the pCa at which *F*_norm_ = 0.5, and *n*_H_ is the Hill coefficient indicating the slope of the force–pCa relation.

### Statistical Analysis

Two-way repeated measures analysis of variance (Two-way RM ANOVA) was performed under GraphPad Prism 4 to test the effects of two factors, the effect of hcTnT-type (hcTnT^R130C^, hcTnT^WT^, and hcTnT^Δ*K*210^) and the effect of fiber length (1.1 *L*_0_ and 1.25 *L*_0_) on each analyzed parameter. Data was subject-matched (fiber-matched) for analyzing the effect of fiber length on parameters that were measured in each individual fiber first at 1.1 *L*_0_ and then at 1.25 *L*_0_. Subject matching was highly indicated by *P* < 0.0001 for each parameter. Significant hcTnT-type-fiber length interaction (*P* < 0.05) in the two-way RM ANOVA indicated dissimilar length change of the parameter among the three hcTnT-types. To probe the cause for significant interaction, *post hoc* analysis was performed using Tukey’s multiple comparison test yielding the *P*-values indicated in the results by ^∗^ for *P* < 0.05, ^∗∗^ for *P* < 0.01, and ^∗∗∗^ for *P* < 0.001. When length affected the parameter with no significant interaction, the significance for length changing the parameter indicated by the subject-matched delta values of the parameter being significantly different from zero was analyzed by Bonferroni post-tests and indicated by # for *P* < 0.05, ## for *P* < 0.01, and ### for *P* < 0.001. All parameter values are given as mean ± SEM (standard error of the mean) of *n* fibers exchanged for each hcTnT-type.

## Results

### Control of Troponin Exchange

The endogenous troponin complex in the left ventricular skinned fibers from guinea pigs was exchanged by exogenous recombinant human cardiac heterotrimeric troponin complex (hcTn) containing the hcTnC and hcTnI wild type subunits and either hcTnT^WT^, hcTnT^R130C^ or hcTnT^Δ*K*210^. The exchange in the fibers of the exogenous hcTn complexes for the endogenous cTn was tested by preparing three samples for each type of recombinant hcTn exchange. Each sample contained two fibers that were subjected to the exchange protocol in the chamber of the mechanical setup under the same conditions as performed for the mechanical measurements and each sample was then quantified for the relative amounts of endogenous and exogenous hcTn (Supplementary Figure S1). The exchange efficiency defined by the ratio of hcTnI per total cTnI (sum of endogenous gcTnI and exogenous hcTnI) was 46 ± 2% for the exchange done with hcTn containing the hcTnT^WT^, 44 ± 1% for the one containing the hcTnT^R130C^ and 48 ± 2% for the one containing the hcTnT^Δ*K*210^ (mean ± SEM of each *n* = 3). There were no significant differences in the efficiencies for the three exchanges. Similar exchange efficiencies have been reported in a previous *in vitro* study of the hcTnT^Δ*K*210^ mutation using exchange of recombinant hcTn for endogenous cTn in permeabilized rabbit cardiac muscle fibers (Morimoto et al., 2002).

### Biomechanical Measurements

**FIGURE 1:**
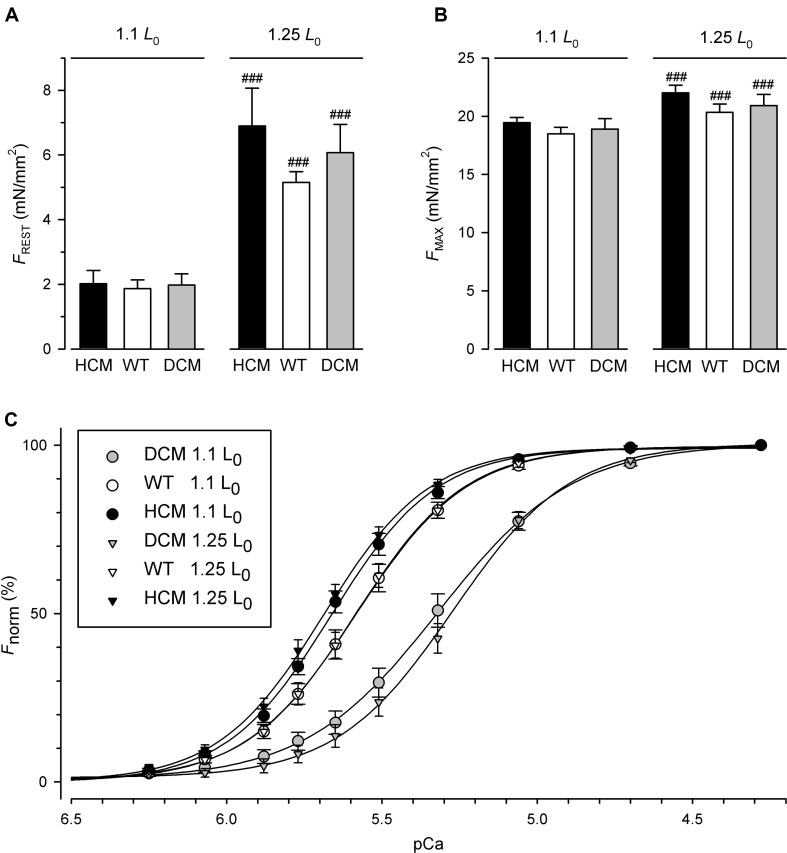
Resting, maximum and calcium-dependent force generation of skinned fibers exchanged for human hcTn containing HCM-associated hcTnT^R130C^ (*n* = 18 fibers), hcTnT^WT^ (*n* = 19 fibers), or DCM-associated hcTnT^Δ*K*210^ (*n* = 19 fibers). **(A)** Resting tension measured in relaxing solution (pCa 7) at short (1.1 *L*_0_) and long (1.25 *L*_0_) fiber length. **(B)** Maximum tension measured at pCa 4.28. **(C)** Force-pCa relations at short fiber length (1.1 *L*_0_) and long fiber length (1.25 *L*_0_). Normalized force is scaled as percentage from 0% for resting tension to 100% for maximum tension. ^###^Indicates highly significant different to 1.1 L0 in paired Bonferroni post tests.

For the force measurements, fibers were prepared from 7 guinea pig hearts. 18 fibers were exchanged for hcTn containing HCM-associated hcTnT^R130C^, 19 fibers contained the hcTnT^WT^ control and 19 fibers contained the DCM-associated hcTnT^Δ*K*210^. Figure 1A shows the resting tension (*F*_REST_) of fibers exchanged with the three different hcTnT at short fiber length (110% of slack length, 1.1 *L*_0_) and after stretching them to long fiber length (125% of slack length, 1.25 *L*_0_). Although the statistical analysis by two-way RM ANOVA indicated strong significant increase of *F*_PASS_ by stretch (*P* < 0.0001) confirmed for each hcTnT-type by Bonferroni post-test (see ### in Figure 1A), there was no significant interaction (*P* = 0.37) between the effects of fiber length and hcTnT-type on *F*_PASS_ (Figure 1A and Table 1). No interaction indicates similar passive mechanical properties of fibers containing the three different types of hcTnT. Similarly to passive tension, two-way RM ANOVA revealed no interaction between the effects of fiber length and hcTnT-type on the maximum tension (*F*_MAX_) during contraction (Figure 1B and Table 1). *F*_MAX_ was strongly significantly increased (*P* < 0.0001) by stretching the fibers. Bonferroni post-tests confirmed the significant effect of stretch on *F*_MAX_ for each hcTnT-type. The results for *F*_MAX_ indicate that the mutations do not alter the maximum force-generating capacity or its length dependence. The values of *F*_PASS_ and *F*_MAX_ are summarized in Supplementary Table S1 (see Supplementary Material) and their statistical analysis in Table 2.

**TABLE 1:** Changes in resting tension, maximum tension, pCa_50_ and *n*_H_ induced by lengthening fibers from 1.1 to 1.25 *L*_0_.

	**hcTnT^R130C^**	**hcTnT^WT^**	**hcTnT^**Δ** K210^**
Delta-*F*_REST_ (mN/mm^2^)	+4.89 ± 0.94^###^	+3.28 ± 0.39^###^	+4.09 ± 0.70^###^
Delta-*F*_MAX_ (mN/mm^2^)	+2.56 ± 0.56^###^	+1.85 ± 0.35^###^	+2.01 ± 0.50^###^
Delta-pCa_50_	+0.030 ± 0.010^#^***	−0.001 ± 0.010	−0.034 ± 0.013^#^
Delta-*n*_H_	−0.06 ± 0.12	−0.02 ± 0.07	+0.22 ± 0.14

Values present mean ± SEM of subject-matched parameter changes, i.e., the parameter changes of the individual fibers exchanged for hcTn containing different hcTnT (hcTnT^*R130C*^ n = 18, hcTnT^*WT*^: n = 19, hcTnT^Δ*K*210^: n = 19). Significant change by lengthening: ^#^P < 0.05, ^##^P < 0.01, ^###^P < 0.001. Significant different to hcTnT^Δ*K*210^: ***P < 0.001.

**TABLE 2:** *P*-values obtained in statistical analysis of the parameters. *F*_pCa’s_ correspond to the normalized force values at the respective pCa.

**Parameter**	**Two-way RM ANOVA**				**Tukey’s multiple comparison**			**Bonferroni post-test**		
	**Interaction**	**hcTnT-type**	**Fiber length**	**Subject match**	**HCM vs. WT**	**HCM vs. DCM**	**DCM vs. WT**	**HCM**	**WT**	**DCM**
*F*_REST_	0.37	0.78	***	***	n.d.	n.d.	n.d.	↑^###^	↑^###^	↑^###^
*F*_ACT_	0.55	0.41	***	***	n.d.	n.d.	n.d.	↑^###^	↑^###^	↑^###^
pCa_50_	***	***	0.81	***	–	***	–	↑^#^	–	*↓*^#^
*n*_H_	0.18	0.37	0.47	***	n.d.	n.d.	n.d.	–	–	–
*F*_pCa6.25_	0.44	0.35	0.25	***	n.d.	n.d.	n.d.	–	–	–
*F*_*Ca6.07*_	***	**	0.67	***	*	***	–	↑^##^	–	*↓*^#^
*F*_*pCa5.88*_	***	***	0.68	***	**	***	*	↑^###^	–	*↓*^###^
*F*_*pCa5.77*_	***	***	0.53	***	–	***	–	↑^##^	–	–
*F*_*pCa5.65*_	**	***	0.50	***	–	**	–	–	–	*↓*^##^
*F*_*pCa5.51*_	**	***	0.63	***	–	**	–	–	–	*↓*^##^
*F*_*pCa5.32*_	***	***	0.11	***	–	***	*	–	–	*↓*^###^
*F*_*pCa5.06*_	0.97	***	0.33	***	n.d.	n.d.	n.d.	–	–	–
*F*_*pCa4.7*_	0.27	***	0.86	***	n.d.	n.d.	n.d.	–	–	–

Two-way RM ANOVA and Tukey’s multiple comparison post-tests were used to test for significant differences among hcTnT-types: *P < 0.05, **P < 0.01, ***P < 0.001. n.d., not determined because of insignificant interaction (P > 0.05). Bonferroni post-tests indicate significant length-dependent changes of parameters for the hcTnT-type: ^#^P < 0.05, ^##^P < 0.01, ^###^P < 0.001; ↑ marks increase, ↓ marks decrease of parameter; – indicates not significant (P > 0.05).

Along with resting tension and maximum tension, the full force-pCa relations were determined before and after lengthening fibers from 1.1 *L*_0_ to 1.25 *L*_0_. Figure 1C illustrates the average force-pCa relations of the three groups of different hcTn-exchanged fibers at short (1.1 *L*_0_, circles) and long fiber length (1.25 *L*_0_, triangles). At short fiber length, the relation of hcTnT^R130C^-exchanged fibers is shifted to the left compared to the relation of the hcTnT^WT^ control, i.e., to higher pCa values or lower [Ca^2+^]. This leftward shift is slightly enhanced upon lengthening the fibers from 1.1 *L*_0_ to 1.25 *L*_0_. Opposite to the HCM mutation, the relation of the fibers containing the DCM-associated hcTnT^Δ*K*210^ is slightly shifted to the right compared to the relation of the control fibers containing the hcTnT^WT^ (Figure 1C). The basal calcium desensitization by the DCM mutation observed at short fiber length is further enhanced by lengthening the fibers to 1.25 *L*_0_.

**FIGURE 2:**
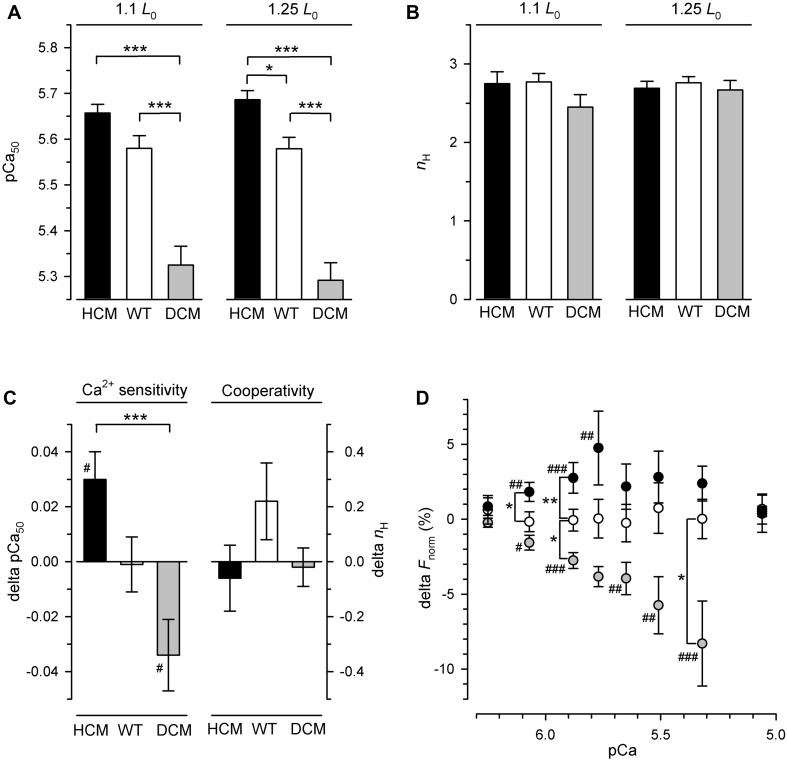
Length-dependent parameters and parameter changes of calcium-dependent force generation of hcTn-exchanged skinned fibers. HCM: fibers containing hcTnT^R130C^ (black circles and bars), WT: fibers containing hcTnT^WT^ (white circles and bars), DCM: fibers containing hcTnT^Δ*K*210^ (gray circles and bars). **(A)** Calcium sensitivity is expressed by the pCa_50_ required for half-maximum increase of calcium-dependent force generation. **(B)** Cooperativity of calcium-dependent force generation is expressed by the Hill coefficient *n*_H_ of force-pCa relations. **(C)** Change of pCa_50_ and Hill coefficient *n*_H_ induced by lengthening the fiber from 1.1 *L*_0_ to 1.25 *L*_0_. **(D)** Change of normalized force at the respective pCa induced by lengthening the fiber from 1.1 *L*_0_ to 1.25 *L*_0_. Change (delta) of parameters in **(C,D)** are calculated by subtracting for each fiber the parameter value at 1.1 *L*_0_ from the parameter value at 1.25 *L*_0_. Significant change of parameter (delta different from zero) is indicated by ^#^*P* < 0.05, ^##^*P* < 0.01, ^###^(*P* < 0.001). Asterisks indicate significant differences *P < 0.05, **P < 0.01, ***P < 0.001 between the hcTnT-types in Tukey’s multiple comparison post-tests. For sake of clarity, only the *P*-values for comparisons between mutants and wild type but not the ones comparing the two mutants were plotted in the subfigure **(D)** (for complete list of *P*-values see Table 2).

To test for statistical significant differences in calcium-dependent force generation among the three types of hcTn exchange and among the two lengths, the force-pCa relation plotted for each individual fiber at each length was fitted by the sigmoidal Hill function (see section “Materials and Methods”) to quantify the mean and variation of pCa_50_ and *n*_H_ as indicators for calcium sensitivity and cooperativity of the calcium-dependent force generation, respectively. Two-way RM ANOVA revealed a highly significant effect (*P* < 0.0001) of the hcTnT-type on the pCa_50_ (Figure 2A, Table 2, and Supplementary Table S1). Most important, there was high interaction (*P* = 0.0009) of the effects of hcTnT-type and fiber length on the pCa_50_ indicating dissimilar lengthening-induced change of pCa_50_ (delta-pCa_50_) among the fibers containing the three different hcTnT-types. In contrast to the strong interaction found for the pCa_50_, there is no interaction (*P* = 0.18), no effect of fiber length (*P* = 0.37) and no effect of hcTnT-type (*P* = 0.47) on the Hill coefficient *n*_H_ in the two-way RM ANOVA (Figure 2B, Table 2, and Supplementary Table S1).

For further analysis of the effect of hcTnT-type on the pCa_50_, the data sets were separated for either short (1.1 *L*_0_) or long (1.25 *L*_0_) fiber length and *post hoc* tested by one-way ANOVA which confirmed the significant effect of hcTnT-type on the pCa_50_ for each of the two lengths (*P* < 0.001). Tukey’s multiple comparison revealed that the DCM mutant highly significantly (*P* < 0.001) decreases Ca sensitivity at both lengths when either compared to the wild type or to the HCM mutant, whereas the HCM mutant increases Ca sensitivity only at long (*P* < 0.05) but not at short fiber length compared to the wild type (Figure 2A and Supplementary Table S1), thus lengthening manifested in an effect of the HCM mutant compared to wild type.

*Post hoc* analysis for searching the reason of the high interaction of hcTnT-type and length effects on the pCa_50_ by Tukey’s multiple comparison yielded highly significant (*P* < 0.001) different delta-pCa_50_ of fibers containing HCM-associated hcTnT^R130C^ compared to fibers containing DCM-associated hcTnT^Δ*K*210^ (Figure 2C and Tables 1, 2). The 95% confidence interval of the delta-pCa_50_ of fibers containing hcTnT^R130C^ was fully positive (+0.002 to +0.058) whereas that of fibers containing hcTnT^Δ*K*210^ was fully negative (−0.062 to −0.007). The significant difference (*P* < 0.05) of the intervals from zero is also reflected by the corresponding Bonferroni post-tests (#-marks in Figure 2C) and indicates that lengthening induced calcium sensitization in fibers containing the HCM mutation whereas lengthening caused calcium desensitization in fibers containing the DCM mutation. The delta-pCa_50_ of fibers containing wild type hcTnT is in-between the delta-pCa_50_ of the fibers containing the mutants and not significantly different to each mutant.

To analyze the effects of hcTnT and length on the calcium-dependent force at sub-maximally activating [Ca^2+^], the normalized force at each pCa was tested by two-way RM ANOVA. Strong significant interaction of hcTnT-type and fiber length effects on normalized force were found for pCa 6.07 (*P* = 0.0002), pCa 5.88 (*P* < 0.0001), pCa 5.77 (*P* = 0.0006), pCa 5.65 (*P* = 0.0022), pCa 5.51 (*P* = 0016), and pCa 5.32 (*P* = 0.006) indicating dissimilarity of lengthening-induced change of normalized force (delta-*F*_norm_) for at least one hcTnT-type at the respective pCa (Table 2). The delta-*F*_norm_ values at submaximal activating pCa are plotted in Figure 2D. Post-tests confirmed that at each of the above pCa, fibers containing the HCM and the DCM mutation differ significantly by at least *P* < 0.01 in their delta-*F*_norm_ (Table 2). At pCa 5.88, all three hcTnT-types differed significantly in delta-*F*_norm_ (*P* < 0.01 for HCM versus WT, *P* < 0.05 for DCM versus WT, and *P* < 0.001 for HCM versus DCM) (Figure 2D and Table 2). In summary, stretching fibers containing the HCM mutation increased calcium sensitivity (pCa_50_) and *F*_norm_ whereas stretching fibers containing the DCM mutation decreased calcium sensitivity and *F*_norm_.

## Discussion

### Basic Effects of the hcTnT Mutations on Calcium Sensitivity

The two mutations R130C and ΔK210 in hcTnT have been associated with autosomal dominant inherited hypertrophic or dilated cardiomyopathy, respectively (Kamisago et al., 2000; Song et al., 2005; Wang et al., 2007). The basal effects of the HCM-associated mutation R130C and of the DCM-associated mutation ΔK210 on calcium sensitivity found in this study resemble the most common phenotype of HCM-mutations and DCM-associated mutations on calcium sensitivity. They are in general agreement with numerous previous studies of HCM- and DCM-associated mutations in hcTnT reporting increase of calcium sensitivity by HCM- and decrease of calcium sensitivity by DCM-associated mutations (Morimoto et al., 2002; Lu et al., 2003, 2013; Venkatraman et al., 2003; Mirza et al., 2005; Robinson et al., 2007; Messer et al., 2016).

The functional consequences of the ΔK210 mutation in hcTnT have been extensively analyzed using *in vitro*, *ex vivo*, and *in vivo* models (Morimoto et al., 2002; Robinson et al., 2002; Venkatraman et al., 2003; Mirza et al., 2005; Du et al., 2007; Robinson et al., 2007; Sfichi-Duke et al., 2010) whereas to the best of our knowledge, there is no functional study for the R130C mutation. A study of knock-in mice expressing cTnT^Δ*K*210^ showed that the decrease of calcium sensitivity was higher in homozygous than in heterozygous cTnT^Δ*K*210^ mice indicating that the calcium desensitization by this mutation increased with the relative amount of mutant protein (Du et al., 2007). The exchange efficiencies in this study of 44–48% are in a good range to mimic the typical co-expression of mutant and wild type hcTnT protein in the heterozygous allelic patients albeit the relative amount of the expression of mutant protein in patients can substantially differ from the theoretical value of 50% (Tripathi et al., 2011). The significant lower calcium sensitivity in absence of significant differences of maximum force and cooperativity of calcium-dependent force generation of fibers containing hcTnT^Δ*K*210^ compared to hcTnT^WT^ found in this study resemble the previously reported effects on these parameters found between fibers isolated from heterozygous cTnT^Δ*K*210^ knock-in and wild type mice (Du et al., 2007). Thus, the *in vitro* exchange of hcTn in cardiac fibers performed in this study qualitatively reproduces the basic functional phenotype found in the *ex vivo* fiber approach (Du et al., 2007). The suitability of the *in vitro* approach to mimic primary effects of mutations on calcium sensitivity together with their diverging basal effects on calcium sensitivity at short fiber length provides a promising starting point for studying their effects on LDA.

### Limitations of the Present Study

An unexpected result in our study was the lack of lengthening-induced change of calcium sensitivity (delta-pCa_50_ = 0) and the low increase of *F*_MAX_ (+10%) in fibers exchanged with hcTn wild type. Most likely the exchange for the recombinant human cardiac troponin complex in the fibers does not restore the LDA of the native cTn. Low LDA might partly result from the dephosphorylated state of cTnI in recombinant cTn (Konhilas et al., 2003). However, previous studies examining effects of cTnT mutation on LDA by exchange of recombinant gcTn into guinea pig cardiac fibers reported delta-pCa_50_ values of +0.1 and increase of *F*_MAX_ by 62–65% after wild type exchange (Reda and Chandra, 2018, 2019). Reda and Chandra (2018, 2019) used recombinant cTn consisting of the guinea pig isoforms while we used recombinant cTn consisting of the human isoforms. Thus, the lacking length-dependent change of pCa_50_ and the low increase of *F*_MAX_ for the wild type control in our study might result from the species-specific difference in the cTn. We chose the human isoforms because the aim of our study was to compare the effects of cTnT mutations related to human cardiomyopathy.

To the best of our knowledge, no functional study of the HCM-associated mutation hcTnT^R130C^ exists so far. We found no difference in calcium sensitivity compared to wild type at short fiber length for this mutation but significant higher calcium sensitivity at long fiber length. Thus, screening the effect of this mutation on calcium sensitivity under basal conditions only would have been negative because the calcium sensitization only became evident under stretch. However, this finding underlines the positive effect of this HCM mutation on LDA.

Finally, we choose rather simple protocol for working at same relative fiber length instead of measuring and adjusting sarcomere length prior activation. Slack length (*L*_0_) were measured prior to hcTn exchange, and biomechanical parameters determined after hcTn exchange at 1.1- and 1.25-fold of that *L*_0_ measured prior exchange. Therefore, all fibers should have similar sarcomere length at *L*_0_ independent of the type of hcTnT. Furthermore, the three different types of hcTnT-exchanged fibers exhibited similar passive and maximum tension and therefore likely adopted similar sarcomere lengths before and during calcium activation in the mechanical experiments.

### Effect of hcTnT Mutations on LDA

The primary aim of the study was to test if HCM and DCM-associated mutations in the human troponin complex exert different effects on the length dependence of mechanical parameters, in particular of calcium sensitivity reflecting LDA. As two-way RM ANOVA analysis indicated no difference in the effects of lengthening on resting and maximum tension for the three type of hcTnT, the HCM-associated R130C and the DCM-associated ΔK210 mutation seem not to alter the basal inhibitory and the maximum regulatory capacity of hcTn. Lengthening changed calcium sensitivity in opposite direction for the two mutations as indicated by the opposite signs of their 95% confidence intervals and the highly significant difference (*P* < 0.001) for their delta-pCa_50_, indicating calcium sensitization by the HCM-associated and calcium desensitization by the DCM-associated mutation. LDA has been also associated to changes in the lattice spacing (McDonald and Moss, 1995; Fuchs and Smith, 2001) and lattice spacing depends on filament charge according to the Donnan potential (Millman and Irving, 1988). Since R130C and ΔK210 both lead to loss of a positive charge in hcTnT, their opposite effects on LDA cannot be explained by filament charge. Instead they likely reflect specific effects of the two sites, 130 and 210 on hcTnT, in the modulation of LDA.

Consistent with our finding of calcium desensitization by the DCM-associated ΔK210 mutation being augmented under stretch, the DCM-associated mutation R174W decreases calcium sensitivity and attenuates the sarcomere length-dependent increase of calcium sensitivity in guinea pig cardiac fibers (Reda and Chandra, 2019). Whether this applies for all DCM-associated mutations in hcTnT needs to be tested in future studies. In any case, it is definitive from previous studies that the mechanism does not apply for all HCM-associated mutations in thin filament proteins and that not all mutations increasing calcium sensitivity enhance LDA. The hypertrophic cardiomyopathy-associated mutation F87L in the central region of cTnT enhances calcium sensitivity but attenuates LDA (Reda and Chandra, 2018) and the RCM-associated mutation hcTnI^R145W^ does not affect LDA although it strongly increases calcium sensitivity (Dvornikov et al., 2016). Thus, modifications of different sites in cTn might exert either positive or negative effects on LDA. Probing the effects of further mutations in Tm and cTn subunits on LDA provides a promising approach to map protein domains involved in LDA for understanding how these proteins integrate the length and the calcium signal for modulating myocardial contraction.

### Possible Contribution of LDA in the Diverging Phenotype of DCM and HCM

Several hypotheses have been formulated to explain the diverging heart phenotypes in HCM versus DCM manifesting from specific mutations within the same protein like cTnT: (1) mutations directly affecting calcium sensitivity (Robinson et al., 2002, 2007), (2) mutations affecting EC coupling or Ca^2+^ homeostasis (Tardiff et al., 2015; Crocini et al., 2016), and (3) mutations interfering with the effect of posttranslational modifications on calcium sensitivity (Sfichi-Duke et al., 2010; Memo et al., 2013; Messer et al., 2016). The systemic development of each of the two diseases in the human is even more complex and highly variable (Maron et al., 2012; Deranek et al., 2019). Studies of human samples revealed that hcTnI is hypo-phosphorylated in myocardial samples from HCM and DCM patients compared to control samples from donor hearts (Hamdani et al., 2008; Sequeira et al., 2013, 2015). It is known that phosphorylation of cTnI by PKA increases lengthening-induced calcium sensitization by increasing the delta-pCa (Konhilas et al., 2003). The lower phosphorylation of cTnI in human patients and animal models for cardiomyopathies compared to control samples could therefore complicate the elucidation of the direct effect of the mutation on LDA. For example, low phosphorylation of hcTnI in HCM-associated patients might reduce LDA and prevent detection of possible increase of LDA by the mutation itself. The simple approach of exchanging recombinant cTn in guinea pig cardiac fibers, like in previous (Mickelson and Chandra, 2017; Reda and Chandra, 2018, 2019) and this study does not include this complication. The direct comparison of HCM and DCM mutant hcTnT found in our study supports the hypothesis that if one excludes posttranslational modulation of myofilaments proteins, HCM- and DCM-associated mutations can increase and decrease LDA, respectively.

Although, our study is in agreement with previous functional studies of the ΔK210 mutation and the prevalent disposition of HCM mutations increasing and DCM mutations decreasing calcium sensitivity, the definite reasons why the ΔK210 mutation results in DCM and the R130C mutation in HCM remain elusive. While typical features of HCM are increased wall thickness, cardiomyocyte disarray, fibrosis and impaired diastolic filling, DCM is characterized by enlarged ventricles, reduced ventricular wall thickness to volume ratio and impaired systolic contraction, i.e., reduced ejection fraction (Garfinkel et al., 2018; Deranek et al., 2019). An interesting hypothesis is that the primary, acute effects of mutations on LDA, in the long term, might contribute to the directionality of the diverging histological and morphological phenotypes of HCM and DCM. Chronically enhanced response to stretch could contribute to strain imbalance of cardiomyocytes, cardiomyocyte disarray and wall thickening in HCM, while impaired contractile response to stretch might lead to overstretched thin cardiomyocytes and enlarged ventricles in DCM. Certainly, the primary effects of the mutations on LDA found in this study are counteractive and not compensatory mechanisms for the primary diastolic dysfunction in HCM and systolic dysfunction in DCM. Regarding the finding that the Frank–Starling mechanism is also impaired in the late-stage heart failure (Schwinger et al., 1994), the effect of the DCM mutation on impairing LDA is expected to be detrimental.

## Data Availability Statement

The datasets generated for this study are available on request to the corresponding author.

## Ethics Statement

Guinea pig were killed for subsequent removal of the heart according to the guidelines approved by the ethical committee of the Landesamt für Natur, Umwelt und Verbraucherschutz Nordrhein-Westfalen (LANUV NRW Leibnizstrasse 10 D-45659 Recklinghausen Germany).

## Author Contributions

MG performed the biomechanical and biochemical experiments and data analysis. AL-D instructed MG in the fiber preparation and the biomechanical experiments. SZ prepared the recombinant protein and instructed MG in the biochemical analysis. GP contributed to the clinical aspects of the discussion. RS designed the study, supervised the experiments, data analysis, and wrote the manuscript.

## Conflict of Interest

The authors declare that the research was conducted in the absence of any commercial or financial relationships that could be construed as a potential conflict of interest.

## References

[B1] Ait Mou Y. Bollensdorff C. Cazorla O. Magdi Y. de Tombe P. P. (2015). Exploring cardiac biophysical properties. *Glob. Cardiol. Sci. Pract.* 2015:10. 10.5339/gcsp.2015.10 26779498 PMC4448074

[B2] Ait-Mou Y. Hsu K. Farman G. P. Kumar M. Greaser M. L. Irving T. C. (2016). Titin strain contributes to the Frank-Starling law of the heart by structural rearrangements of both thin- and thick-filament proteins. *Proc. Natl. Acad. Sci. U.S.A.* 113 2306–2311. 10.1073/pnas.1516732113 26858417 PMC4776536

[B3] Arteaga G. M. Palmiter K. A. Leiden J. M. Solaro R. J. (2000). Attenuation of length dependence of calcium activation in myofilaments of transgenic mouse hearts expressing slow skeletal troponin I. *J. Physiol.* 526(Pt 3) 541–549. 10922006 10.1111/j.1469-7793.2000.t01-1-00541.x PMC2270032

[B4] Crocini C. Ferrantini C. Scardigli M. Coppini R. Mazzoni L. Lazzeri E. (2016). Novel insights on the relationship between T-tubular defects and contractile dysfunction in a mouse model of hypertrophic cardiomyopathy. *J. Mol. Cell. Cardiol.* 91 42–51. 10.1016/j.yjmcc.2015.12.013 26714042 PMC4767219

[B5] de Tombe P. P. Mateja R. D. Tachampa K. Ait Mou Y. Farman G. P. Irving T. C. (2010). Myofilament length dependent activation. *J. Mol. Cell. Cardiol.* 48 851–858. 10.1016/j.yjmcc.2009.12.017 20053351 PMC2854194

[B6] Deranek A. E. Klass M. M. Tardiff J. C. (2019). Moving beyond simple answers to complex disorders in sarcomeric cardiomyopathies: the role of integrated systems. *Pflugers Arch.* 471 661–671. 10.1007/s00424-019-02269-0 30848350 PMC6476637

[B7] Du C.-K. Morimoto S. Nishii K. Minakami R. Ohta M. Tadano N. (2007). Knock-in mouse model of dilated cardiomyopathy caused by troponin mutation. *Circ. Res.* 101 185–194. 17556660 10.1161/CIRCRESAHA.106.146670

[B8] Dvornikov A. V. Smolin N. Zhang M. Martin J. L. Robia S. L. de Tombe P. P. (2016). Restrictive cardiomyopathy troponin I R145W mutation does not perturb myofilament length-dependent activation in human cardiac sarcomeres. *J. Biol. Chem.* 291 21817–21828. 27557662 10.1074/jbc.M116.746172 PMC5076848

[B9] Farman G. P. Gore D. Allen E. Schoenfelt K. Irving T. C. de Tombe P. P. (2011). Myosin head orientation: a structural determinant for the Frank-Starling relationship. *Am. J. Physiol. Heart Circ. Physiol.* 300 H2155–H2160. 10.1152/ajpheart.01221.2010 21460195 PMC3119094

[B10] Fuchs F. Smith S. H. (2001). Calcium, cross-bridges, and the Frank-Starling relationship. *News Physiol. Sci.* 16 5–10. 11390938 10.1152/physiologyonline.2001.16.1.5

[B11] Garfinkel A. C. Seidman J. G. Seidman C. E. (2018). Genetic pathogenesis of hypertrophic and dilated cardiomyopathy. *Heart Fail. Clin.* 14 139–146. 10.1016/j.hfc.2017.12.004 29525643 PMC5851453

[B12] Hamdani N. Kooij V. van Dijk S. Merkus D. Paulus W. J. Remedios C. D. (2008). Sarcomeric dysfunction in heart failure. *Cardiovasc. Res.* 77 649–658. 18055579 10.1093/cvr/cvm079

[B13] Hanft L. M. Korte F. S. McDonald K. S. (2008). Cardiac function and modulation of sarcomeric function by length. *Cardiovasc. Res.* 77 627–636. 18079105 10.1093/cvr/cvm099

[B14] Kalyva A. Parthenakis F. I. Marketou M. E. Kontaraki J. E. Vardas P. E. (2014). Biochemical characterisation of Troponin C mutations causing hypertrophic and dilated cardiomyopathies. *J. Muscle Res. Cell Motil.* 35 161–178. 10.1007/s10974-014-9382-0 24744096

[B15] Kamisago M. Sharma S. D. DePalma S. R. Solomon S. Sharma P. McDonough B. (2000). Mutations in sarcomere protein genes as a cause of dilated cardiomyopathy. *N. Engl. J. Med.* 343 1688–1696. 11106718 10.1056/NEJM200012073432304

[B16] Konhilas J. P. Irving T. C. Wolska B. M. Jweied E. E. Martin A. F. Solaro R. J. (2003). Troponin I in the murine myocardium: influence on length-dependent activation and interfilament spacing. *J. Physiol.* 547 951–961. 12562915 10.1113/jphysiol.2002.038117 PMC2342721

[B17] Korte F. S. Feest E. R. Razumova M. V. Tu A.-Y. Regnier M. (2012). Enhanced Ca^2+^ binding of cardiac troponin reduces sarcomere length dependence of contractile activation independently of strong crossbridges. *Am. J. Physiol. Heart Circ. Physiol.* 303 H863–H870. 10.1152/ajpheart.00395.2012 22865385 PMC3469702

[B18] Kruger M. Pfitzer G. Stehle R. (2003). Expression and purification of human cardiac troponin subunits and their functional incorporation into isolated cardiac mouse myofibrils. *J. Chromatogr. B Analyt. Technol. Biomed. Life Sci.* 786 287–296. 12651025 10.1016/s1570-0232(02)00763-8

[B19] Li A. Y. Stevens C. M. Liang B. Rayani K. Little S. Davis J. (2013). Familial hypertrophic cardiomyopathy related cardiac troponin C L29Q mutation alters length-dependent activation and functional effects of phosphomimetic troponin I^∗^. *PLoS One* 8:e79363. 10.1371/journal.pone.0079363 24260207 PMC3832503

[B20] Linke W. A. (2018). Titin gene and protein functions in passive and active muscle. *Annu. Rev. Physiol.* 80 389–411. 10.1146/annurev-physiol-021317-121234 29131758

[B21] Lu Q.-W. Morimoto S. Harada K. Du C.-K. Takahashi-Yanaga F. Miwa Y. (2003). Cardiac troponin T mutation R141W found in dilated cardiomyopathy stabilizes the troponin T-tropomyosin interaction and causes a Ca^2+^ desensitization. *J. Mol. Cell. Cardiol.* 35 1421–1427. 14654368 10.1016/j.yjmcc.2003.09.003

[B22] Lu Q.-W. Wu X.-Y. Morimoto S. (2013). Inherited cardiomyopathies caused by troponin mutations. *J. Geriatr. Cardiol.* 10 91–101. 10.3969/j.issn.1671-5411.2013.01.014 23610579 PMC3627712

[B23] Maron B. J. Maron M. S. Semsarian C. (2012). Genetics of hypertrophic cardiomyopathy after 20 years: clinical perspectives. *J. Am. Coll. Cardiol.* 60 705–715. 10.1016/j.jacc.2012.02.068 22796258

[B24] Marston S. Zamora J. E. (2020). Troponin structure and function: a view of recent progress. *J. Muscle Res. Cell Motil.* 41 71–89. 10.1007/s10974-019-09513-1 31030382 PMC7109197

[B25] Marston S. B. Hodgkinson J. L. (2001). Cardiac and skeletal myopathies: can genotype explain phenotype? *J. Muscle Res. Cell Motil.* 22 1–4. 11563546 10.1023/a:1010355716511

[B26] McDonald K. S. Moss R. L. (1995). Osmotic compression of single cardiac myocytes eliminates the reduction in Ca^2+^ sensitivity of tension at short sarcomere length. *Circ. Res.* 77 199–205. 7788878 10.1161/01.res.77.1.199

[B27] Memo M. Leung M.-C. Ward D. G. dos Remedios C. Morimoto S. Zhang L. (2013). Familial dilated cardiomyopathy mutations uncouple troponin I phosphorylation from changes in myofibrillar Ca^+^ sensitivity. *Cardiovasc. Res.* 99 65–73. 23539503 10.1093/cvr/cvt071

[B28] Messer A. E. Bayliss C. R. El-Mezgueldi M. Redwood C. S. Ward D. G. Leung M.-C. (2016). Mutations in troponin T associated with Hypertrophic Cardiomyopathy increase Ca^2+^-sensitivity and suppress the modulation of Ca^2+^-sensitivity by troponin I phosphorylation. *Arch. Biochem. Biophys.* 601 113–120. 27036851 10.1016/j.abb.2016.03.027 PMC4909753

[B29] Mickelson A. V. Chandra M. (2017). Hypertrophic cardiomyopathy mutation in cardiac troponin T (R95H) attenuates length-dependent activation in guinea pig cardiac muscle fibers. *Am. J. Physiol. Heart Circ. Physiol.* 313 H1180–H1189. 10.1152/ajpheart.00369.2017 28842439

[B30] Millman B. M. Irving T. C. (1988). Filament lattice of frog striated muscle. Radial forces, lattice stability, and filament compression in the A-band of relaxed and rigor muscle. *Biophys. J.* 54 437–447. 3264728 10.1016/S0006-3495(88)82977-1 PMC1330343

[B31] Mirza M. Marston S. Willott R. Ashley C. Mogensen J. McKenna W. (2005). Dilated cardiomyopathy mutations in three thin filament regulatory proteins result in a common functional phenotype. *J. Biol. Chem.* 280 28498–28506. 15923195 10.1074/jbc.M412281200

[B32] Morimoto S. Lu Q. W. Harada K. Takahashi-Yanaga F. Minakami R. Ohta M. (2002). Ca^2+^-desensitizing effect of a deletion mutation Delta K210 in cardiac troponin T that causes familial dilated cardiomyopathy. *Proc. Natl. Acad. Sci. U.S.A.* 99 913–918. 11773635 10.1073/pnas.022628899 PMC117405

[B33] Neulen A. Blaudeck N. Zittrich S. Metzler D. Pfitzer G. Stehle R. (2007). Mn2+-dependent protein phosphatase 1 enhances protein kinase A-induced Ca^2+^ desensitisation in skinned murine myocardium. *Cardiovasc. Res.* 74 124–132. 17321507 10.1016/j.cardiores.2007.01.019

[B34] Reda S. M. Chandra M. (2018). Cardiomyopathy mutation (F88L) in troponin T abolishes length dependency of myofilament Ca^2+^ sensitivity. *J. Gen. Physiol.* 150 809–819. 10.1085/jgp.201711974 29776992 PMC5987878

[B35] Reda S. M. Chandra M. (2019). Dilated cardiomyopathy mutation (R174W) in troponin T attenuates the length-mediated increase in crossbridge recruitment and myofilament Ca^2+^ sensitivity. *Am. J. Physiol. Heart Circ. Physiol.* 317 H648–H657. 10.1152/ajpheart.00171.2019 31373515

[B36] Robinson P. Griffiths P. J. Watkins H. Redwood C. S. (2007). Dilated and hypertrophic cardiomyopathy mutations in troponin and alpha-tropomyosin have opposing effects on the calcium affinity of cardiac thin filaments. *Circ. Res.* 101 1266–1273. 17932326 10.1161/CIRCRESAHA.107.156380

[B37] Robinson P. Mirza M. Knott A. Abdulrazzak H. Willott R. Marston S. (2002). Alterations in thin filament regulation induced by a human cardiac troponin T mutant that causes dilated cardiomyopathy are distinct from those induced by troponin T mutants that cause hypertrophic cardiomyopathy. *J. Biol. Chem.* 277 40710–40716. 12186860 10.1074/jbc.M203446200

[B38] Schwinger R. H. Bohm M. Koch A. Schmidt U. Morano I. Eissner H. J. (1994). The failing human heart is unable to use the Frank-Starling mechanism. *Circ. Res.* 74 959–969. 8156643 10.1161/01.res.74.5.959

[B39] Sequeira V. Najafi A. Wijnker P. J. M. Dos Remedios C. G. Michels M. Kuster D. W. D. (2015). ADP-stimulated contraction: a predictor of thin-filament activation in cardiac disease. *Proc. Natl. Acad. Sci. U.S.A.* 112 E7003–E7012. 10.1073/pnas.1513843112 26621701 PMC4687530

[B40] Sequeira V. Wijnker P. J. M. Nijenkamp L. L. A. M. Kuster D. W. D. Najafi A. Witjas-Paalberends E. R. (2013). Perturbed length-dependent activation in human hypertrophic cardiomyopathy with missense sarcomeric gene mutations. *Circ. Res.* 112 1491–1505. 10.1161/CIRCRESAHA.111.300436 23508784 PMC3675884

[B41] Sfichi-Duke L. Garcia-Cazarin M. L. Sumandea C. A. Sievert G. A. Balke C. W. Zhan D.-Y. (2010). Cardiomyopathy-causing deletion K210 in cardiac troponin T alters phosphorylation propensity of sarcomeric proteins. *J. Mol. Cell. Cardiol.* 48 934–942. 10.1016/j.yjmcc.2010.01.005 20079745 PMC2854196

[B42] Solzin J. Iorga B. Sierakowski E. Gomez Alcazar D. P. Ruess D. F. Kubacki T. (2007). Kinetic mechanism of the Ca^2+^-dependent switch-on and switch-off of cardiac troponin in myofibrils. *Biophys. J.* 93 3917–3931. 17704185 10.1529/biophysj.107.111146 PMC2099212

[B43] Song L. Zou Y. Wang J. Wang Z. Zhen Y. Lou K. (2005). Mutations profile in Chinese patients with hypertrophic cardiomyopathy. *Clin. Chim. Acta* 351 209–216. 15563892 10.1016/j.cccn.2004.09.016

[B44] Stehle R. Kruger M. Scherer P. Brixius K. Schwinger R. H. G. Pfitzer G. (2002). Isometric force kinetics upon rapid activation and relaxation of mouse, guinea pig and human heart muscle studied on the subcellular myofibrillar level. *Basic Res. Cardiol.* 97 (Suppl. 1) I127–I135. 12479246 10.1007/s003950200041

[B45] Tardiff J. C. Carrier L. Bers D. M. Poggesi C. Ferrantini C. Coppini R. (2015). Targets for therapy in sarcomeric cardiomyopathies. *Cardiovasc. Res.* 105 457–470. 10.1093/cvr/cvv023 25634554 PMC4402369

[B46] Tripathi S. Schultz I. Becker E. Montag J. Borchert B. Francino A. (2011). Unequal allelic expression of wild-type and mutated beta-myosin in familial hypertrophic cardiomyopathy. *Basic Res. Cardiol.* 106 1041–1055. 10.1007/s00395-011-0205-9 21769673 PMC3228959

[B47] Venkatraman G. Harada K. Gomes A. V. Kerrick W. G. L. Potter J. D. (2003). Different functional properties of troponin T mutants that cause dilated cardiomyopathy. *J. Biol. Chem.* 278 41670–41676. 12923187 10.1074/jbc.M302148200

[B48] Wang S.-X. Zou Y.-B. Fu C.-Y. Song L. Wang H. Wang J.-Z. (2007). [Family hypertrophic cardiomyopathy caused by a 14035c > t mutation in cardiac troponin T gene]. *Zhonghua Yi Xue Za Zhi* 87 371–374. 17456375

[B49] Willott R. H. Gomes A. V. Chang A. N. Parvatiyar M. S. Pinto J. R. Potter J. D. (2010). Mutations in Troponin that cause HCM, DCM AND RCM: what can we learn about thin filament function? *J. Mol. Cell. Cardiol.* 48 882–892. 10.1016/j.yjmcc.2009.10.031 19914256

[B50] Zhang X. Kampourakis T. Yan Z. Sevrieva I. Irving M. Sun Y.-B. (2017). Distinct contributions of the thin and thick filaments to length-dependent activation in heart muscle. *eLife* 6:e24081. 10.7554/eLife.24081 28229860 PMC5365314

