# Supplementary material for: Hypertrophic and Dilated Cardiomyopathy-Associated Troponin T Mutations R130C and ΔK210 Oppositely Affect Length-Dependent Calcium Sensitivity of Force Generation
Source: Front Physiol. 2020 Jun 3;11:516. doi: 10.3389/fphys.2020.00516 (PMC7283609; doi:10.3389/fphys.2020.00516)
Supplement: Supplementary file 1 [file Data_Sheet_1.pdf]

SUPPLEMENTARY MATERIAL

Hypertrophic and dilated cardiomyopathy-associated Troponin T Mutations R130C and ΔK210 Oppositely Affect Length-Dependent Calcium Sensitivity of Force Generation

Marcel Groen[1], Alfredo Jesus López-Dávila[2], Stefan Zittrich[3], Gabriele Pfitzer[4], Robert Stehle[3,*]

[1] Department of Neurology and Neurogeriatry, Johannes Wesling Medical Center, Ruhr University Bochum, Minden, Germany

[2] Department of Molecular and Cell Physiology, Hannover Medical School, Hannover, Germany

[3] Institute of Vegetative Physiology, University of Cologne, Köln, Germany

[4] Institute of Neurophysiology, University of Cologne, Köln, Germany

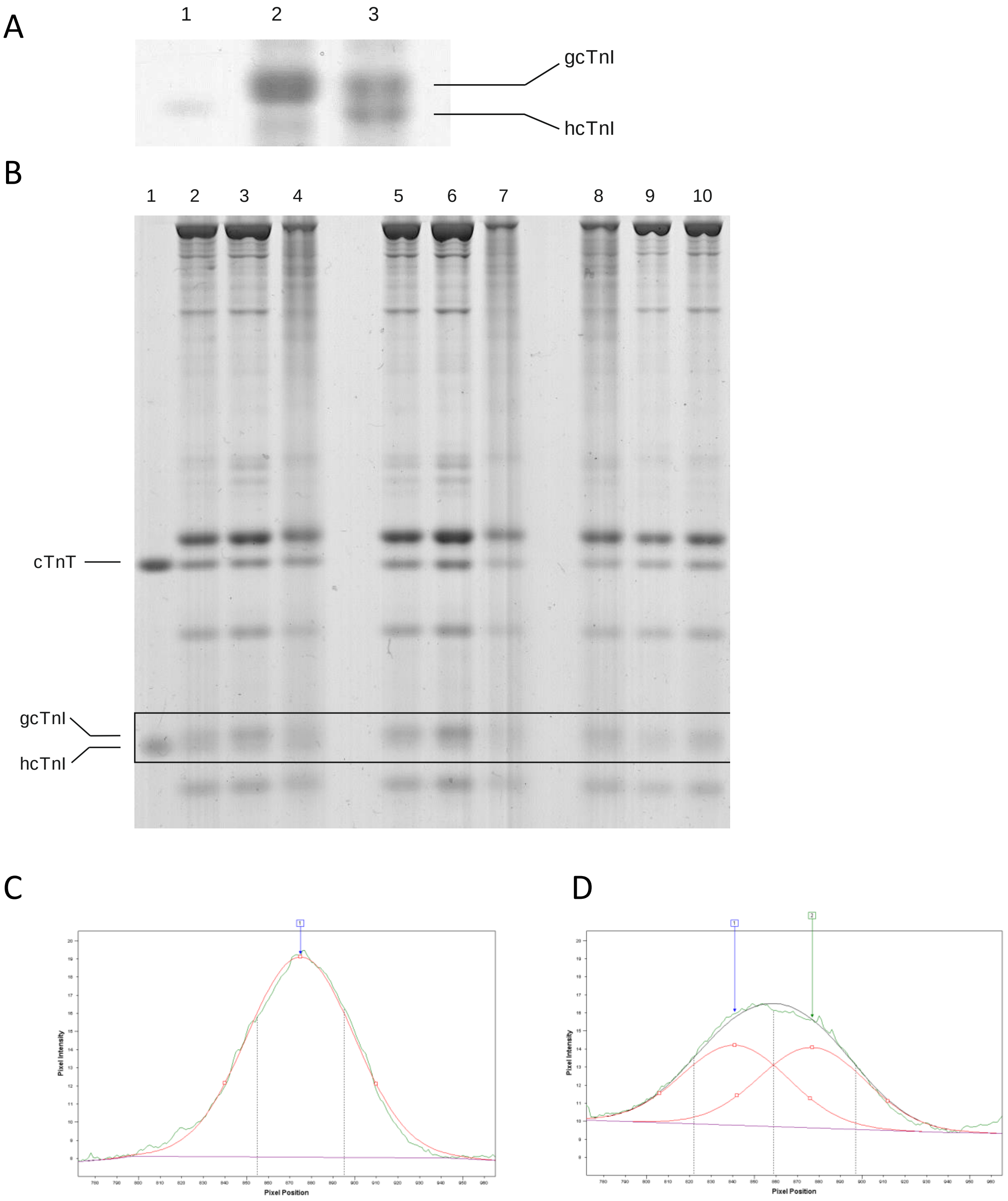

Figure S1 Quantification of the exchange of endogeneous guinea pig cTn (gcTn) for recombinant human cTn (hcTn). **(A)** Commassie-R250-stained 12.5 % SDS-PAGE showing the separation of guinea pig cardiac troponin I (gcTnI) and human cardiac troponin I (hcTnI). Lane 1: hcTn, lane 2: left ventricular fibers of guinea pig. Lane 3: left ventricular fibers of guinea pig exchanged for hcTn. Recombinant hcTnI contains one less amino acid and can be separated from the native gcTnI. **(B)** Commassie-R250-stained 12.5 % SDS-PAGE performed to determine the exchange efficiencies in the present study. Lane 1 contained the recombinant hcTn as marker. Each of the lanes 2-10 contained two left ventricular guinea pig fibers exchanged for heterotrimeric hcTn that consisted of hcTnC, hcTnI and either $hcTnT^{WT}$ (lanes 2-4), $cTnT^{R130C}$ (lanes 5-7) or $hcTnT^{\Delta K210}$ (lanes 8-10). **(C) and (D)** Quantification of the gel scan show in *(B)* for content of gcTnI and hcTnI in Phoretix-1. The green lines show

the intensity profiles integrated over the rectangular part marked in *(B)*. The profiles were fit by the sum of a background function (pink lines) and Gaussian peak functions of same half-widths (red lines). **(C)** The half-width was determined by fitting a single Gaussian peak to the intensity profile of hcTnI (lane 1 in *(B)*). This half-width was set as fixed value for fitting all cTnI bands in the lanes 2-10. **(D)** The green line shows the intensity profile integrated from lane 3 in *(B)*. The black line shows the best fit to the profile presenting the sum of the background function (pink line) and the two Gaussians (red lines). Individual intensities of gcTn and hcTnI are obtained from the area of the left and right Gaussian peak, respectively.

	$hcTnT^{R130C}$		$hcTnT^{WT}$		$hcTnT^{\Delta K210}$	
Parameter	1.1 L_0	1.25 L_0	1.1 L_0	1.25 L_0	1.1 L_0	1.25 L_0
F_{REST} (mN/mm^2)	2.02 ± 0.41	6.90 ± 1.17 ###	1.87 ± 0.27	5.15 ± 0.33 ###	1.98 ± 0.34	6.07 ± 0.88 ###
F_{MAX} (mN/mm^2)	19.44 ± 0.45	22.00 ± 0.66 ###	18.48 ± 0.56	20.33 ± 0.72 ###	18.90 ± 0.90	20.91 ± 0.97 ###
pCa_{50}	5.657 ± 0.019 §§§	5.686 ± 0.020 * §§§	5.580 ± 0.028	5.579 ± 0.025	5.325 ± 0.038 ***	5.292 ± 0.038 ***
n_H	2.75 ± 0.15	2.69 ± 0.09	2.77 ± 0.11	2.76 ± 0.08	2.45 ± 0.16	2.67 ± 0.12

Table S1 Resting tension (F_{REST}), maximum tension (F_{MAX}), calcium sensitivity indicated by pCa_{50} and cooperativity of calcium activation indicated by Hill coefficient n_H of fibers exchanged for hcTn containing different hcTnT ($hcTnT^{R130C}$ *n*=18, $hcTnT^{WT}$: *n*=19, $hcTnT^{\Delta K210}$: n=19) at short fiber length (1.1 L_0) and long fiber length (1.25 L_0). Values present means ± SEM. Significant change by lengthening indicated in Bonferroni post-tests: $^{\#}P<0.05$, $^{\#\#}P<0.01$, $^{\#\#\#}P<0.001$. Significant different to WT (*$P<0.05$, ***$P<0.001$) or significant different to $hcTnT^{\Delta K210}$ ($^{§§§}P<0.001$) in Tukey´s multiple comparison post-tests.
